# Counterfeit phosphodiesterase type 5 inhibitors pose significant safety risks

**DOI:** 10.1111/j.1742-1241.2009.02328.x

**Published:** 2010-03

**Authors:** G Jackson, S Arver, I Banks, V J Stecher

**Affiliations:** 1 Cardiac Department, St. Thomas HospitalLondon, UK; 2 Andrology Centre, Karolinska University HospitalStockholm, Sweden; 3 Men's Health, Leeds Metropolitan UniversityLeeds, UK; 4 Global Sexual Health, Pfizer IncNew York, NY, USA

## Abstract

Counterfeit drugs are inherently dangerous and a growing problem; counterfeiters are becoming increasingly sophisticated. Growth of the counterfeit medication market is attributable in part to phosphodiesterase type 5 inhibitor (PDE5i) medications for erectile dysfunction (ED). Millions of counterfeit PDE5is are seized yearly and account for the bulk of all counterfeit pharmaceutical product seizures. It has been estimated that up to 2.5 million men in Europe are exposed to illicit sildenafil, suggesting that there may be as many illegal as legal users of sildenafil. Analysis of the contents of counterfeit PDE5is shows inconsistent doses of active pharmaceutical ingredients (from 0% to > 200% of labelled dose), contaminants (including talcum powder, commercial paint and printer ink) and alternative ingredients that are potentially hazardous. In one analysis, only 10.1% of samples were within 10% of the labelled tablet strength. Estimates place the proportion of counterfeit medications sold over the Internet from 44% to 90%. Of men who purchase prescription-only medication for ED without a prescription, 67% do so using the Internet. Counterfeit PDE5is pose direct and indirect risks to health, including circumvention of the healthcare system. More than 30% of men reported no healthcare interaction when purchasing ED medications. Because > 65% actually had ED, these men missed an opportunity for evaluation of comorbidities (e.g. diabetes and hypertension). Globally, increased obstacles for counterfeiters are necessary to combat pharmaceutical counterfeiting, including fines and penalties. The worldwide nature of the counterfeit problem requires proper coordination between countries to ensure adequate enforcement. Locally, physicians who treat ED need to inform patients of the dangers of ordering PDE5is via the Internet.

**Review Criteria**

We performed an exhaustive search for articles concerning counterfeit medication using multiple sources, including PubMed, government and organisational websites and legal journals. We focused the search on phosphodiesterase type 5 inhibitor (PDE5i) medications to provide a description of the scope of the problem and the safety risks associated with counterfeit PDE5i medication.

**Message for the Clinic**

Counterfeit drugs are inherently dangerous and are a growing problem; industry growth is attributable in part to PDE5i medication for erectile dysfunction (ED). Men often use the Internet to obtain PDE5i medication, a practice associated with direct risks (e.g. receiving counterfeit medication) and indirect risks (e.g. circumvention of the healthcare system). Physicians who treat ED should inform patients of the dangers of ordering PDE5is via the Internet.

## Introduction

A recent report in the *New England Journal of Medicine* chronicled an outbreak of hypoglycaemia in patients using counterfeit sexual enhancement drugs (1). Glyburide, a powerful drug used for the treatment of diabetes, was found to be a contaminant in counterfeit tadalafil and herbal preparations for the treatment of erectile dysfunction (ED) (1). Of the 150 non-diabetic patients admitted to hospitals in Singapore, seven patients were comatose as a result of severe neuroglycopenia; four patients subsequently died (1).

This case is just one of many that report the dangers inherent in counterfeit drugs. Other examples include two women in Argentina who died after being given injections of a counterfeit iron preparation to treat anaemia; another woman survived but had a 26-week premature baby (2). The prevalence of counterfeit artesunate, an antimalarial drug, has been increasing in Southeast Asia. Without the required active ingredients, patients have died, and the cause of death was often mistakenly attributed to drug resistance (3). In Bangladesh, at least 51 children died in an outbreak of diethylene glycol poisoning of paracetamol syrup, with many more victims suspected (4). Diethylene glycol contamination of paracetamol syrup was responsible for an estimated 192,000 deaths in 2002 (5).

Although death is the most extreme consequence of counterfeit medication, other dangers of counterfeits are more widespread. Counterfeit drugs may contain excessive or ineffective levels of active ingredients, contaminants, or inactive or dangerous ingredients. For example, tap water has been found as the only ingredient in counterfeit neomycin eye drops and meningococcal vaccine, counterfeit ampicillin consisting of turmeric and counterfeit contraceptive pills made of wheat have been reported, and counterfeit antibiotics and snake antivenom (in addition to antimalarials) have been found to lack active ingredients (6).

### What is a counterfeit drug and how big is the problem?

The definition of counterfeit drugs varies between countries, but the World Health Organization (WHO) defines counterfeit medicines as those that ‘are deliberately and fraudulently mislabelled with respect to identity or source: their quality is unpredictable as they may contain the wrong amount of active ingredients, wrong ingredients or no active ingredients…[they are] manufactured in clandestine laboratories with no possibility of control (2).’

The prevalence of counterfeit drugs appears to be increasing; however, the scale of an illicit industry is difficult to determine. Counterfeit seizures in the European Union increased 384% between 2005 and 2006 (7), with a further 51% increase in 2007 (8). Customs officials noted that counterfeits were becoming more difficult to distinguish from their genuine counterparts (9).

In the United States, the number of investigations by the Food and Drug Administration (FDA) into drug fraud in 2003 was almost four times that in 2000 (10). Additionally, the FDA also noted increased complexity and organisation of the counterfeit medication industry (10). The FDA has seen an 800% increase in the number of new counterfeit cases between 2000 and 2006 (11). The Center for Medicine in the Public Interest estimates that the global sales of counterfeit drugs will reach $75 billion in 2010, a 92% increase from 2005 (11,12).

In most developed countries with effective regulatory systems and market control, it is estimated that < 1% of sales are counterfeit, although many indications point to an increasing prevalence of counterfeits (13). Overall, approximately 10–30% of medications in developing markets may be counterfeit (13). Some countries of Africa, parts of Asia and parts of Latin America have areas where more than 30% of the medicines on sale can be counterfeit; many former Soviet republics have a proportion close to 20% (13). Given the scope of the problem, the number of people who may be exposed to adverse events with counterfeit drug use is substantial.

### How are phosphodiesterase type 5 inhibitors related to counterfeit drugs?

The growth in counterfeit medications is partly because of the advent of effective oral medications for ED (14); sildenafil (Viagra®; Pfizer Inc, New York, NY, USA) and other phosphodiesterase type 5 inhibitors [PDE5is, including tadalafil (Cialis®; Eli Lilly and Company, Indianapolis, IN, USA) and vardenafil (Levitra®; Bayer HealthCare, Wayne, NJ, USA)] are the drugs most likely to be counterfeited. According to EU counterfeit seizures, PDE5is were the most commonly counterfeited class of product in Europe (15); they are a prime target for counterfeiting because of their high cost and the embarrassment associated with the underlying condition (14).

During the 5 years spanning from 2004 to 2008, 35.8 million counterfeit sildenafil tablets were seized in Europe. In 2004, law officers seized 10.6 million counterfeit sildenafil tablets, which was seven times the number of all other counterfeited Pfizer products combined (16). In 2006, 2.5 million counterfeit sildenafil tablets were seized in the European Union and accounted for 96% of the counterfeit Pfizer products seized (16). In 2007, 3.4 million counterfeit sildenafil tablets were seized (16).

Two different analyses estimated the market size for illicit sildenafil (16). From these analyses, it was estimated that 0.6–2.5 million men in Europe could be exposed to illicit sildenafil. For comparison, IMS Health (Norwalk, CT) estimates the number of users of legal sildenafil in the European Union to be 2.5 million in 2006.

### What is in counterfeit phosphodiesterase type 5 inhibitors?

**Figure 1:**
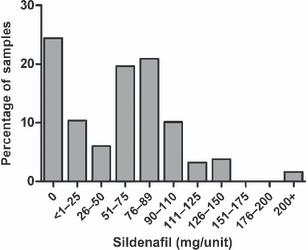
Sildenafil dosage of counterfeit products (*n*= 316) analysed by Pfizer Samples were labelled as ‘Viagra 100 mg’

At least some active ingredient was present in the counterfeit Viagra that was seized by the Medical Control Agency of the United Kingdom (40–100% of the labelled dosage) (17). A portion (*n*= 2383) of the pharmaceutical agents seized by authorities (including customs, law enforcement and health agencies) for suspicion of being counterfeit Viagra were forwarded to Pfizer for analysis between 2005 and 2009 (18). Spectral analysis determined authenticity of samples and high-performance liquid chromatography determined purity and active pharmaceutical ingredient (API) concentration in a subset of samples. Inconsistent dosage of API (0% to > 200% of the indicated strength of sildenafil, Figure 1), as well as impurities or alternative ingredients with potentially hazardous substances were found.

Only 10.1% of samples labelled ‘Viagra 100 mg’ were within 10% of the advertised tablet strength (Figure 1). A sample of counterfeit Viagra from Hungary was found to contain only amphetamine, a stimulant with potent adverse effects. Counterfeit Viagra seized in the United Kingdom contained caffeine and bulk lactose, with only 30 mg of the advertised 100 mg of sildenafil citrate. Talcum powder, commercial-grade paints, and active ingredients for completely different pharmaceuticals have been found in a subset of seized illicit Viagra samples. Paracetamol and metronidazole, an antiprotozoal/anti-infective, have been found in counterfeit Viagra. Metronidazole has significant adverse effects when combined with alcohol; overdose of paracetamol can cause potentially fatal liver damage, with alcohol intake and alcoholism significantly increasing this risk (19,20). Printer ink has been used to colour tablets blue. Additionally, large quantities of unidentified impurities have been found in counterfeit tablets.

The Dutch National Institute for Public Health and Environment (RIVM) analysed 370 samples of illegally sold Viagra collected from 2000 to 2004 and found that only 10 were genuine (21). Often, sildenafil was present, but in lower amounts than declared. In addition, other pharmaceuticals, such as amphetamine, clomiphene, chloramphenicol, dipyrone, fluoxetine, tadalafil, yohimbine, gamma-amino butyric acid, caffeine, l-arginine, indigotin and quinine were found in seized samples (21).

In a follow-up survey by three governmental laboratories in the Netherlands (RIVM, the Nederlands Forensisch Instituut, and the Douane Laboratorium), an increased number of imitation erectogenic products (defined as illegal drug products that do not look like the genuine medicine, but by their name or claim strongly suggest an erectogenic effect) was found, from 50% in 2000 to 2004 to 65% in 2005 to 2006 (22). The most commonly found imitation was Kamagra, a sildenafil-containing medication that is manufactured by a legitimate Indian pharmaceutical company; however, this compound is not licensed in the European Union or the United States. Sildenafil was found in 69% of the 95 uncontrolled (i.e. counterfeit and imitation) erectogenic products, including Kamagra. Tadalafil, which had represented only 2% of samples in the previous analysis, accounted for 25% of samples and vardenafil accounted for 6% (22).

The US National Association of State Boards of Pharmacy (NABP) ordered several different medications, including Cialis from 13 suspicious websites selling prescription-only medication without a prescription (23). The FDA analysed the active ingredients and found wide variation in the amount present, ranging from none to far exceeding the level allowed in US medications (23). Lilly, the drug manufacturer, performed a regulatory analysis on the products obtained by NABP and found that they did not meet the company's US standards (23).

‘Herbal’ or ‘dietary’ supplements are also marketed for sexual dysfunction, and consumers may believe these products to be harmless (24). When 17 commercial formulations were analysed, eight contained approved PDE5is or related compounds, including sildenafil, tadalafil, vardenafil, hydroxyhomosildenafil, thiosildenafil and thiomethisosildenafil (25). An analysis of a ‘100% natural product’ for ED that was purchased over the counter and directly from the manufacturer using the Internet revealed that the three samples analysed contained an average of 55 mg of sildenafil per capsule (26).

### What are the dangers of counterfeit phosphodiesterase type 5 inhibitors?

#### Direct risks

The presence of unknown pharmaceutically active ingredients and/or impurities may lead to undesirable and serious adverse events or even death. Dosage variability and dosage mislabelling may lead to accidental overdose. Additionally, incorrect or incomplete descriptions of product composition create a substantial risk for drug–drug interactions. Minimal or incorrect guidance about contraindications may lead to serious adverse events. Because inert ingredients provide no treatment benefit, patients remain untreated and may be discouraged from seeking additional help because they believe that medication does not work for them.

#### Indirect risks

Unsupervised use of PDE5is, particularly by men with ED and common comorbid conditions (e.g. hypertension, dyslipidaemia and diabetes) may occur. Because these diseases carry a significant risk of morbidity or mortality, men who omit a physician visit to discuss their ED also lose an opportunity to address potential underlying conditions. This is a concern because men with health problems have been shown to be less likely than women to consult a physician (27).

Using drugs that are not what they are supposed to be provides no basis for determining drug efficacy or providing guidance for future use. Importantly, addressing adverse events of an unknown product is difficult and perhaps dangerous, as illustrated by the glyburide contamination incident described earlier. Statistics for adverse events and fatalities resulting from counterfeit products appear low relative to the amount of illicit PDE5is available. However, suppliers work outside any regulatory authority, which makes defects and adverse drug reactions difficult to recognise, monitor or recall (28).

### The role of the Internet

Counterfeit PDE5is are closely associated with the Internet. The majority of counterfeit PDE5is in the United States are thought to enter the market via fraudulent websites (29). In Europe, the Experts on the Operation of European Conventions in the Penal Field estimate that 44% of sildenafil sold over the Internet is counterfeit (30). Between 4500 and 15,000 websites sell PDE5is and other substances used to treat ED (29), although other estimates place this number at 500,000 (31). Sites receive 12.9 million visitors per month and sell approximately 2.3 million tablets per month, most without a prescription (29).

Evidence indicates that the Internet is generally unsafe for the purchase of medications. The European Alliance for Access to Safe Medicines (EAASM), a pan-European patient safety initiative committed to promoting the exclusion of counterfeit and substandard medicines from the supply chain, examined the general safety of online pharmacies (32). Of more than 100 online pharmacies identified and assessed, 93.8% had no named, verifiable pharmacist; 90.3% did not require a prescription for prescription-only medications; 84.5% did not have a physical location; and 78.8% had a website that violated intellectual property (32). When two orders of 18 different prescription-only medications (including PDE5is) were placed online without a prescription, 38% were genuine branded medicine (although 16% of these were illegal non-EU imports and 33% had no patient information leaflet). The remaining 62% were substandard or counterfeit medicine; 68% of these were ‘generic’ imitations and 32% were branded (32).

Similar findings by other groups echo these startling statistics. WHO has suggested that medicines purchased over the Internet from sites that conceal their physical address are counterfeit ≥ 50% of the time (2). The president of America's Watchdog, a consumer advocacy group, has stated that 90% of all medicines sold over the Internet are fake (33).

Despite these risks, many persons are using the Internet to buy medication. According to a study of 935 men aged ≥ 35 years in major UK cities, 1 in 10 men has purchased a prescription-only medication without a prescription, and 50% of those men do so via the Internet (14,34). The percentage of men purchasing prescription-only medications without a prescription via the Internet increases to 67% when considering ED medications specifically (14).

Additionally, a survey of French men (*n*= 301) found that even among men with valid prescriptions for a PDE5i, 12% obtained their ED treatment via the Internet (16). In another study conducted in March 2007 in several European countries, six of 51 men in Spain and the United Kingdom reported using a non-registered and uncontrolled ‘generic’ version of sildenafil, called Kamagra, which is usually sold over the Internet (16).

### Phosphodiesterase type 5 inhibitors ordered via the Internet are associated with risky behaviour

Circumvention of primary care is the very first risk associated with Internet-ordered medication. An Internet-based observational analysis of the sources used by men in the United Kingdom, Germany and France who obtained PDE5is within the last 12 months (*n*= 373) was conducted (35). The sources of purchase were either within the healthcare system (HCS), defined as any supply through retail pharmacy, prescription or a free sample from a physician; or outside the HCS, defined as Internet or mail-order pharmacy without a prescription or uncontrolled sources such as non-pharmacy Internet sources or from a friend or partner.

**Figure 2:**
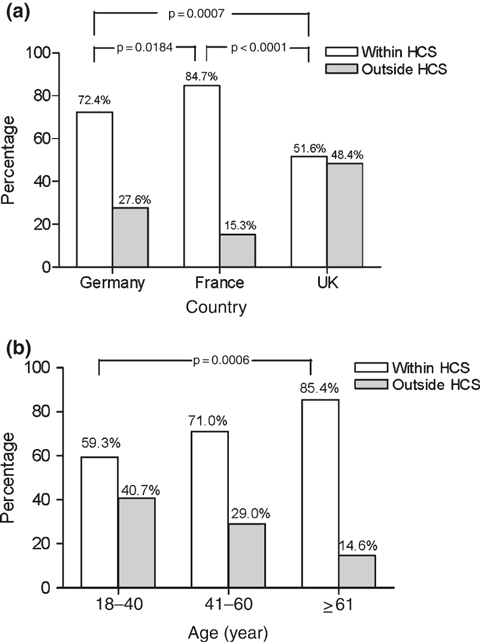
Distribution of men who interacted within or outside the healthcare system (HCS) to obtain phosphodiesterase type 5 inhibitors by (a) country and (b) age. A chi-square test was used to test associations, statistical tests were two-tailed, and the Bonferroni-Holm sequential step-down method was applied to control the overall alpha level, set at 0.05

Overall, 30.6% of participating men had no interaction with the HCS. Significant discrepancies were evident between countries: in the United Kingdom, 48.4% of men used sources outside the HCS, whereas 27.6% of German men and 15.3% of French men used sources outside the HCS (Figure 2). Age discrepancies also were evident: 40.7% of men 18–40 years of age used sources outside the HCS, whereas 29.0% of those 41–60 years and 14.6% of those ≥ 61 years did so.

Of men who reported using sources outside the HCS, 72% reported having ED (43% mild, 25% moderate, 4% severe), which, as discussed above, may be associated with common cardiovascular risk factors (36). Additionally, the probability of hearing about known contraindications of ED treatments was likely reduced. Furthermore, men who reported using sources outside the HCS were more likely to purchase a ‘generic’ imitation PDE5i than those who used sources within the HCS (35).

**Figure 3:**
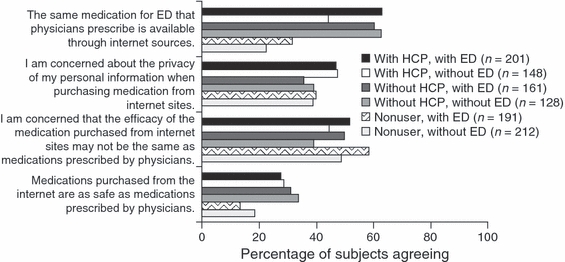
Attitudes towards phosphodiesterase type 5 inhibitors obtained via the Internet. ED, erectile dysfunction; HCP, healthcare professional. Reproduced from Schnetzler et al. (37) with permission from Blackwell Publishing Ltd.

In a larger analysis following this pilot study, 10.5% of almost 12,000 men in an Internet-based observational study conducted in the United Kingdom, Germany and Italy reported PDE5i use in the previous 6 months (37). Almost one-third (32.3%) of men obtained their PDE5i without healthcare professional (HCP) interaction. HCP interaction was defined as having a prescription for a PDE5i, having a sample of PDE5i from a physician, or buying the PDE5i in a retail pharmacy; non-HCP sources included friends, the Internet and partners or other acquaintances. More men without HCP interaction than with HCP interaction used the Internet to purchase their medication (51.6% vs. 27.6%). More than 60% of men who bypassed HCP interaction to obtain their PDE5i believed they would get the same prescription-only medication via the Internet (Figure 3). The majority of men were not concerned about the efficacy of medicines ordered via the Internet, although most generally felt that medicines sold via the Internet were less safe than those prescribed by a physician (Figure 3). Of men bypassing HCP interaction, 65.5% genuinely had ED. Thus, a significant number of men missed the opportunity for evaluation of possible morbidities that coexist with ED.

Substantive evaluations are not offered by the ‘cyberdoctors’ used by many Internet sites to create prescriptions (38). It is unclear whether these cyberdoctors are real people or merely computer programs; their credentials may not exist (39). Some sites have disclaimers stating that physicians may not reside in the same country as the patient or the pharmacy. Alarmingly, patients with obvious contraindications to sildenafil use have been prescribed the drug (40).

The circumvention of the HCS is not the only risk associated with Internet purchase of PDE5is. Men who buy PDE5is via the Internet appear to use more pills than those who do not. A survey of purchasing channels for men in the United Kingdom, France and Germany revealed that men who obtained sildenafil from a pharmacy or directly from a physician consumed an average of 13 pills within the last 12 months, whereas those purchasing through the Internet consumed 20 pills and those taking unauthorised generics consumed 29 pills in the same time period (16). Thus, patients purchasing through the Internet have increased risk of exposure to counterfeit PDE5is. Phosphodiesterase type 5 inhibitors are associated with intentional abuse or recreational use in certain subpopulations, and surveys indicate that the Internet is a source for recreational PDE5is (41). Further, sources of information about abusing PDE5is, such as methods to enhance or exaggerate expected effects, are readily found on the Internet (41).

### What can be done to combat counterfeit phosphodiesterase type 5 inhibitors and other drugs?

The WHO has stated, ‘Counterfeit medicines are a threat to our communities and must be stopped. This problem is not isolated to a handful of countries; it is present everywhere and it is gaining momentum. It is not a problem of one person, it is a problem of all people. It is not a problem of one country, it is a problem of all nations (42).’

To combat the problem, there is a general consensus that increased obstacles for counterfeiters are necessary to reduce risks associated with counterfeit drugs as well as their sale via the Internet (2,6,11,15,43–47). However, a clear worldwide consensus on what constitutes a counterfeit drug is lacking. Activities associated with counterfeit drug manufacturing and distribution may be illegal in some countries but not in others. For example, India and China do not recognise European patent authority and can legally manufacture generic drugs that would be illegal to produce in the United Kingdom (48). However, the United Kingdom legally permits importation of products for personal use that are licensed in the country of origin, including those ordered via the Internet (5,48). From January to July of 2005, > 250 kg of sildenafil citrate was exported from India to European destinations; 1 kg can produce approximately 14,000 counterfeit Viagra tablets (49). When sold at market price, 2000% profits can be realised, which is approximately 10 times the profitability of heroin (49). This type of profit comes with comparatively little risk: in the United Kingdom, it has been reported that the prison sentence and fine for counterfeiting a T-shirt with a trademarked logo can be greater than for counterfeiting a medicine (11).

Because of the worldwide nature of the counterfeiting problem, proper coordination between countries is absolutely essential (17). However, many countries lack the resources and/or political willpower to prioritise an anticounterfeiting agenda. Countries interested in prosecuting counterfeiters are hindered by a lack of international cooperation. For example, in the United States, only counterfeiting operations that take place on US soil or involve directly misleading regulatory authorities are prosecuted; diplomatic obstacles and varying laws between countries are some of the many hindrances to action (28). Jurisdiction within the United States has been described as confusing and overlapping (50). The UK's Medicines and Healthcare Products Regulatory Agency continually monitors Internet sites that sell prescription-only medications, but is only able to take action against sites based in the United Kingdom (51). Overseas sites are referred to the regulatory body of their country of operation (51).

Implementation of fines and penalties is often proposed as a solution, but in practice, collection is far from simple (52). Resources and dedication to enforcement of tough penalties are needed to deter the dangerous practice of counterfeiting medications. Unfortunately, it will be difficult to define success because the extent of the current level of risk is unknown (53).

For ED specifically, the threat of Internet-associated purchase of PDE5is is not fully recognised. Lack of awareness by HCPs and consumers has been cited by the WHO's International Medical Products AntiCounterfeiting Taskforce as a key factor that makes counterfeiting possible (2), a sentiment echoed by others involved in investigating counterfeit medications (47). In the study of 935 UK men described earlier, 32% regarded taking prescription-only medication without a prescription as a low or neutral risk. However, 60% admitted that knowing that their medication was counterfeit would affect the likelihood of purchase, even though most were unsure of the specific adverse effects such a purchase may pose (14,34).

The EAASM believes that informing patients of possible risks associated with Internet purchasing of medications will increase awareness (32). Campaigns organised by governments and industry and directed at consumers have been launched. Physicians who treat ED should inform patients of the dangers of ordering PDE5is via the Internet as part of a multipronged strategy to combat counterfeiting.
